# Blunt Talk on “Blunts”: The Increasingly Popular Tobacco Product That Is Potentially Exacerbating Tobacco-Related Health Disparities

**DOI:** 10.1007/s11606-024-08980-8

**Published:** 2024-09-23

**Authors:** Joshua I. Sanchez, Reece S. Fong, Katherine Hampilos, Ziva D. Cooper, Holly R. Middlekauff

**Affiliations:** 1 Department of Medicine, Division of Cardiology, and Department of Physiology, UCLA David Geffen School of Medicine, Los Angeles, CA USA; 2 UCLA Center for Cannabis and Cannabinoids, Jane and Terry Semel Institute for Neuroscience and Human Behavior, Department of Psychiatry and Biobehavioral Sciences, and Department of Anesthesiology and Perioperative Medicine, UCLA David Geffen School of Medicine, Los Angeles, CA USA

## Abstract

A “blunt” is a hollowed-out cigar/cigarillo from which much of the loose tobacco has been removed, and the remaining tobacco wrapper filled with cannabis. Although blunts contain significant levels of tobacco/nicotine, they are often treated as if they were exclusive cannabis products and omitted from surveys of tobacco products. Whereas the prevalence of virtually all other tobacco products is on the decline in the USA, available data suggest that the prevalence of blunt smoking is not — and in fact, it may be increasing. Blunts are most frequently used by people who self-identify as Black. As a result of misperceptions and perhaps biases, there is a dearth of scientific investigation, hence knowledge, surrounding the health effects associated with blunt smoking. Co-use of tobacco and cannabis has been reported to have additive and even synergistic adverse health effects. Lack of investigations into the health effects of tobacco products most frequently used by Black people may contribute to tobacco-related health disparities. We argue that the scientific and public health communities must treat blunts as the potentially lethal tobacco product that they are, studying their prevalence and use patterns, and investigating their adverse health effects, both short and long term.

## SCOPE OF THE PROBLEM

A “blunt” is a hollowed-out cigar or cigarillo from which much of the loose tobacco has been removed, and the remaining tobacco wrapper has been filled with cannabis.^1^ Although blunts contain tobacco/nicotine,^2^ the scientific and public health communities have treated them as if they were exclusive cannabis products, typically omitting them from surveys of tobacco products, as well as from public health messaging.^3–5^ Additionally, and perhaps consequently, blunts are often mistakenly believed to deliver only cannabis by the people who use them.^6^ Whereas the prevalence of virtually all other tobacco products, including combusted tobacco and even electronic cigarettes is on the decline in the USA, available data suggest that the prevalence of blunt smoking is not — and in fact, it may be increasing.^5,7–9^ Blunts are most frequently used by people who self-identify as Black,^1,5,10^ and current (past 30-day) blunt use has been reported to be as high as 18.5% in this vulnerable population.^5,7,10–12^ As a result of misperceptions and perhaps biases, there is a dearth of scientific investigation, hence knowledge, surrounding the health effects associated with blunt smoking.^5,13^ There is reason for concern, however, since co-use of tobacco and cannabis has been reported to have additive and even synergistic adverse health effects.^14^ The health effects of blunt smoking, a unique form of co-use, in which tobacco and cannabis are *co-administrated*, that is, inhaled simultaneously, have not been studied systematically, despite the increasing popularity of blunts. Lack of investigations into the health effects of tobacco products most frequently used by Black people may have contributed to tobacco-related health disparities.^13,15^ We argue that the scientific and public health communities must treat blunts as the potentially lethal tobacco product that they are, studying, with improved measurement tools,^16^ their prevalence and use patterns, including the influence of the tobacco industry on these factors, and investigating their adverse health effects, both short and long term. Only then will critical data be available with which to inform widespread public health messaging, and effective regulation.

## WHO USES BLUNTS?

Perhaps more is known about the origin of blunt making than the current patterns and prevalence of blunt smoking. Blunts purportedly arose from the hip-hop youth subculture in the 1980s and 1990s, and to this day are most frequently used by young people, especially males, who self-identify as Black.^1,5,10^ Data reflecting prevalence and patterns of blunt smoking over years are largely unavailable for the simple reason that they have not been systematically collected. Although indisputably a tobacco product, blunts have not been a focus of tobacco control efforts and, in fact, are often omitted from surveys of tobacco product use, contributing to current knowledge gaps in their prevalence and physiologic effects. It was only in 2019 that the National Youth Tobacco Survey first included the prevalence of Cigar, Little Cigar and Cigarillo (CLCC) use in their report, and uncovered that Black and Hispanic youth were disproportionately susceptible to these products.^6^ Unfortunately, the prevalence of blunt use was not surveyed, an important omission. Only two national surveillance programs include questions about co-administration of cannabis and tobacco, the National Survey on Drug Use and Health (NSDUH) and the Population Assessment of Tobacco and Health (PATH) study, and these lack precision. For example, the PATH survey does not allow for distinction between blunt smokers, and blunt smokers who use cigarettes, unmodified cigars, or cigarillos in rapid succession (“blunter chasers”). Most reports that include blunts are based on targeted populations, limited to specific geographic areas, race/ethnicities, or age groups. The best data we have, based largely on these two national surveys and the qualitative and observational reports, suggest that blunt prevalence is greatest in non-Hispanic Black youth and young adults, and the prevalence of past month blunt usage ranges from 4.8 to 18.5%.^5,7,9–12,17^

## INFLUENCE OF THE TOBACCO INDUSTRY

It is impossible to discuss the popularity of blunt smoking without acknowledging the influence of the tobacco industry. Marketing (or “co-marketing”) tactics for cigarillos highlight features that promote and facilitate their use for cannabis delivery, therefore using the appeal of cannabis to increase tobacco product sales.^18^ For example, the tobacco wrappers of certain brands of cigarillos popular for blunt-making feature a line of perforation, allowing easy splitting for tobacco removal, which is replaced by cannabis.^19^ Cigarillos called “Splitarillos” advertise this feature with the image of a zipper on the packaging.^18^ Additionally, cigarillos may have a cannabis-related product name or label, e.g., “Juicy bluntzilla”.^18^ These are just a few examples how the tobacco industry is taking advantage of the appeal of cannabis to youth to promote ongoing sales of tobacco products.

The inclusion and promotion of flavorings in cigars and cigarillos is another such tactic. Flavored cigarettes, except menthol cigarettes (also most popular with people who self-identify as Black), were banned by the Family Smoking Prevention and Tobacco Control Act in 2009. Cigars and cigarillos were not included in this flavor ban, and accordingly, cigarillo wrappers often feature menthol, candy, and fruit flavors. Unsurprisingly, flavors have been identified by young people as the leading reason why they smoke cigarillos.^19^ It is anticipated that there will be a federal ban on flavors on all tobacco products including cigars, cigarillos, and blunt wrappers. Tobacco-free blunt wraps are now widely available, and although the FDA is well-positioned to regulate them, it has not yet explicitly claimed this authority.^20^ Accordingly, the alarm bell has been sounded^20^ that the tobacco industry will use the tobacco-free blunt wraps, which are already marketed under names reminiscent of cigars (“…rillo”), therefore fillable with tobacco leaf, to skirt the anticipated federal flavor bans on all tobacco products, perpetuating the appeal of tobacco products to youth.

Finally, in contrast to cigarettes, for which pack sizes are regulated, no minimal pack size is mandated for cigar products, and cigarillos are sold in small resealable packs of one to three, rendering them much more affordable to cash-strapped young people. Cigarillos are also more likely to be sold in neighborhoods with a greater proportion of Black or low-income residents, with reports of targeted marketing in these communities.^21,22^ Although somewhat controversial,^23–25^ it appears that regulation of flavored cigarillos and wrappers, along with cigar taxing and increased minimum pack sizes, especially if implemented federally, could help contribute to a decrease in cigarillo and blunt use nationwide.^26,27^

## WHAT DO WE KNOW ABOUT THE TOBACCO/NICOTINE CONTENT IN BLUNTS?

Also attributable to lack of scientific investigation, little is known about the tobacco/nicotine exposure from blunt smoking. The lone investigation that we could find reported the nicotine content in five different cigar wrappers used for blunt smoking.^2^ Nicotine was quantifiable in all five wrappers and ranged from 1.2 to 6.0 mg per cigar. Although less than the nicotine content in a tobacco cigarette, which may contain 10–15 mg of nicotine, it is not trivial. It has been estimated that 5 mg of nicotine daily is enough to establish a tobacco use disorder.^28^ Further, this value does not account for tobacco left in the wrapper during blunt preparation. Finally, it is important to recognize that the relationship between tobacco smoking burden and cardiovascular risk is not linear.^29^ Even minimal daily smoking levels, for example one to three cigarettes per day, confer significant cardiovascular risk, not dissimilar compared to the risk conferred by smoking one to three packs per day.^29^ Accordingly, even a small amount of tobacco/nicotine would be expected to have significant adverse health effects. This lack of fundamental knowledge regarding the range of nicotine exposures associated with blunt smoking is further evidence of the absence of scientific investigation — and perhaps interest — into the tobacco product most frequent used by Black people.

## DO BLUNTS SERVE AS A REVERSE GATEWAY?

Blunts have been described as a “reverse gateway,” defined as a substance that precedes or prompts the initiation of tobacco use. Weekly or more cannabis use during adolescence has been associated with increased risk of tobacco use in adulthood, thereby serving as a reverse gateway to tobacco-only use.^30^ Similarly, blunt use has also been hypothesized to function as a reverse gateway, in part due to the known synergistic effects of both tobacco and cannabis on a primed endocannabinoid system that can subsequently lead to greater potential for nicotine use.^14,31,32^ Studies have reported that adults and adolescents who had used a blunt within the past year were up to four times more likely to start using cigarettes and up to six times more likely to use any kind of cigar compared to those who abstained from blunts and other forms of cannabis use.^31–33^ Whereas we do not know if this increased risk of initiation of tobacco-only products equates to their long-term use, the potential role of blunts as a reverse gateway highlights the urgent need for scientific data to inform potentially life-or-death decision-making in people who are choosing to initiate blunt smoking, thus preventing use of combusted tobacco-only products in this vulnerable population.

## ADVERSE EFFECTS OF CO-ADMINISTRATION OF CANNABIS AND TOBACCO

Co-use of cannabis and tobacco has been associated with greater likelihood of cannabis use disorder (CUD) and poorer cannabis cessation outcomes relative to cannabis-only use.^2,34,35^ Blunt use has also been associated with increased substance use severity and increased difficulty in reducing both cannabis and tobacco use. For example, population studies have shown that past-year blunt smoking is linked to an overall increase in the risk of developing CUD.^33^ Lastly, cannabis users who prefer blunts and attempt to quit have a higher risk of relapse and/or increasing tobacco consumption to mitigate nicotine withdrawal.^36^

Other health effects of cannabis and tobacco co-use are understudied and largely unknown. Both substances have been linked to adverse cardiovascular outcomes, including arrhythmias and acute coronary ischemia, with co-use hypothesized to contribute to even greater adverse effects on the cardiovascular system compared to either compound alone.^37,38^ Co-use of cannabis and tobacco has also been associated with increased likelihood of psychosocial and behavioral problems as well as diminished lung function compared with cannabis use alone.^34,39–41^ However, research on the health effects of blunt use, which represents a unique form of co-use, that is, *simultaneous co-administration* of cannabis and tobacco, is scarce. A recent systematic review on co-administration revealed that most of the published literature comes from qualitative and descriptive studies, with no studies reporting the long-term health consequences of using blunts or other co-administered cannabis and tobacco products.^5^ Additional research is needed not only to understand the health effects of co-use, but also to determine optimal intervention strategies for cessation of both substances.

## WHY DO SUCH GLARING GAPS IN KNOWLEDGE EXIST?

Although the reasons for the paucity of investigations into the health effects of this common form of cannabis and tobacco inhalation are unknown and likely complex, an important factor is the type of surveillance data available to accurately estimate the prevalence of blunt use. Challenges with gathering data related to blunt use include (1) whether blunts should be categorized as a tobacco product and therefore included in tobacco surveillance studies, or if they should be included only in cannabis use studies; (2) misperceptions of product content by users, as illustrated by the 2015–2019 NSDUH study, in which the proportion of people reporting blunt use exceeded the proportion who self-reported any cannabis use;^6,16,42^ (3) misperceptions that may be compounded by miscommunication, since investigators may be unfamiliar with the current lexicon/jargon, and thus use terminology not recognized by the user, consequently eliciting inaccurate, negative responses;^16^ (4) and biases towards vulnerable populations in whom disparities in research have long existed. All of these factors, and likely others, have resulted in the current dearth of knowledge surrounding the health effects of blunt smoking, thereby potentially contributing to longstanding tobacco-related health disparities.^43^

## THE PROPOSAL

We propose taking a multitude of steps to understand the health effects of blunt use, with the goal of vastly reducing, or preferably, eliminating, their use. First, there must be an increase in funding for investigations into patterns and prevalence of blunt use, as well as their short- and long-term health effects. To acquire accurate data regarding who is using blunts and how often, we must also correctly classify blunts as both a tobacco and cannabis product and develop survey tools using language recognized by the people who use these products. Further, these surveys must reflect the complexity, evolution, and diversity of the cannabis market such as tobacco-free wraps, and of cannabis-use behaviors, such as blunt chasing. In acquiring these data from surveys and longitudinal studies, it will be critical to develop widespread public relations campaigns to educate people who are already using blunts, and those who are contemplating initiation, regarding the health effects of blunt smoking. In addition, research into the role of tobacco industry practices that may contribute to blunt use is necessary; these practices include sales of single cigarillos, perforated wrappers, flavored tobacco-wrappers, and tobacco-free wrappers. Understanding if and how regulation of these products can curb use will be a critical step in safeguarding youth and reducing tobacco-related health disparities.
